# Impact of Roasting Temperature on Antioxidant Activities and Characterization of Polyphenols in Date Seed Beverages From Different Cultivars

**DOI:** 10.1111/1750-3841.70242

**Published:** 2025-05-07

**Authors:** Linghong Shi, Kashif Ghafoor, Claudia Gonzalez Viejo, Sigfredo Augusto Fuentes Jara, Farhad Ahmadi, Hafiz A. R. Suleria

**Affiliations:** ^1^ School of Agriculture, Food and Ecosystem Sciences, Faculty of Science The University of Melbourne Parkville Victoria Australia; ^2^ Digital Agriculture, Food and Wine Group, School of Agriculture, Food and Ecosystem Sciences, Faculty of Science The University of Melbourne Parkville Victoria Australia

**Keywords:** coffee alternative, date palm seed, functional foods, phenolic profiling, phytochemicals

## Abstract

This experiment aimed to analyze the antioxidant capacity and composition of polyphenolic compounds from date seed beverage produced from eight date palm cultivars subjected to three roasting temperatures (180°C, 200°C, and 220°C) from light roasting to dark roasting. Total phenolic content in date seed beverages at light roasting ranged from 4.98 to 14.09 mg GAE/g, higher than that of the medium roasting (3.66–8.65 mg GAE/g) and dark roasting intensity (1.66–6.33 mg GAE/g). Date seed beverages produced from lightly roasted seeds had higher antioxidant capacity than those roasted at medium and dark levels. Using LC‐ESI‐QTOF‐MS/MS, a total of 69 polyphenolic compounds were detected, classified into three groups: 17 phenolic acids, 40 flavonoids, and 12 other phenolic compounds. Our findings demonstrated a decrease in phenolic content as date seed roasting intensity increased from light to dark roasting, accompanied by variations in both phenolic composition and antioxidant capacity across cultivars.

## 1 Introduction

The cultivation of date palm represents a critical agricultural resource that substantially supports the economies of its growing regions (Chao and Krueger 2007). According to Statista (2023), global date production has experienced significant expansion over the past few decades, reaching 9.66 million tons in 2021. The increase in production has enhanced food security in regions where dates are cultivated, while also establishing dates as a significant export product in international markets. However, the rapid growth in production has brought with it challenges related to waste management, particularly concerning date seeds, which are often discarded (Muñoz‐Tebar et al. 2023).

Previous research has demonstrated that date seeds are a rich source of bioactive compounds, including antioxidants and dietary fiber (Ghafoor et al. 2022). This necessitates further research into the development of methods to maximize the nutritional potential of date seeds and valorize this underutilized resource. In particular, roasted date seed powders have been used to prepare coffee‐like beverages in the Arabian region, where this practice has a long‐standing cultural tradition (Ghnimi et al. 2017). It has been reported that the roasting process significantly impacts the phenolic content and antioxidant properties of date seeds (Babiker et al. 2020), with a large variation reported across cultivars (Khatib et al. 2022).

Despite growing interest, existing research has primarily focused on Middle Eastern cultivars and a limited range of roasting conditions, resulting in a limited understanding of how date seeds from other regions respond to roasting in terms of their bioactive compound profile and functional properties. Moreover, studies have shown that both cultivar and roasting temperature can significantly influence the antioxidant capacity and phenolic profile of date seeds. For instance, phenolic degradation and structural transformations, such as dehydration and isomerization, may occur during roasting, resulting in reduced levels of phenolic and flavonoid compounds (Babiker et al. 2020). These findings highlight the need for systematic studies that explore how different roasting levels affect the bioactive potential of different date cultivars.

To address this knowledge gap, this study was designed to assess the antioxidant properties and to identify phenolic compounds of date seed powders from eight Australian‐grown date palm cultivars roasted at three different temperatures. In contrast to previous research that has largely focused on a limited combination of cultivars and roasting intensity, this investigation aims to provide a comprehensive comparison of different cultivars and roasting intensities. General colorimetric assays were employed for an initial assessment of total phenolic, flavonoid, and condensed tannin contents, as well as antioxidant capacity. In addition, a detailed profiling of polyphenolic compounds was conducted using LC‐ESI‐QTOF‐MS/MS analysis. This information is expected to contribute to optimizing the roasting conditions for maximizing the nutritional value of different date cultivars and to potentially providing a foundation for sustainable valorization of this underutilized waste.

## 2 Material and Methods

### 2.1 Reagents and Chemicals

The following chemicals were obtained from Sigma‐Aldrich (Castle Hill, NSW, Australia): vanillin, gallic acid, Folin–Ciocalteu reagent, L‐ascorbic acid, catechin, hexahydrate quercetin, aluminum chloride, DPPH, ABTS, TPTZ, and alizarin. Sulfuric acid (98%) was sourced from RCI Labscan (Rongmuang, Thailand). Methanol, ferric chloride, glacial acetic acid, acetonitrile, hydrated sodium acetate, and hydrochloric acid were purchased from Thermo Fisher Scientific Inc. (Scoresby, VIC, Australia). Sodium carbonate (in anhydrous form) was purchased from Chem‐Supply Pty Ltd. (Adelaide, Australia).

### 2.2 Preparation of Experimental Samples

**FIGURE 1:**
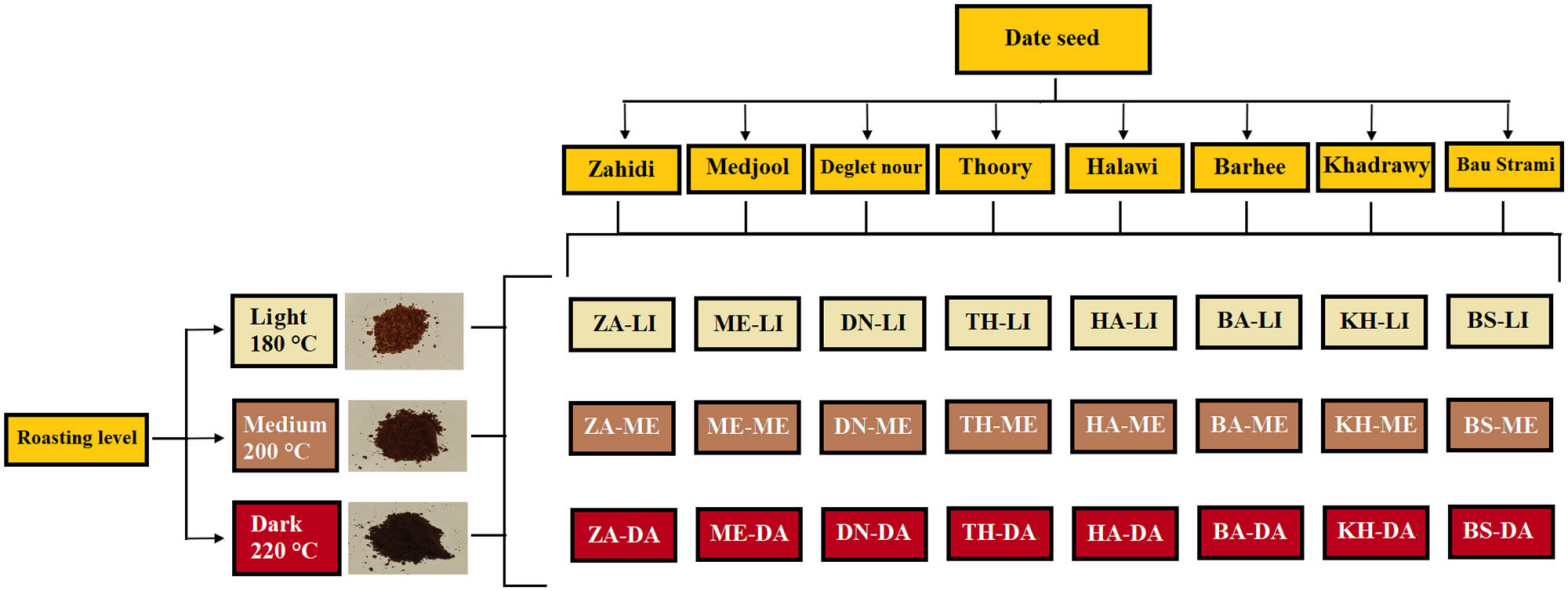
Sample abbreviations of date seed coffee‐like beverage at different roasting levels.

Eight cultivars of dates (Zahidi, Medjool, Deglet nour, Thoory, Halawi, Barhee, Khadrawy, and Bau Strami) at the mature (*Tamar*) ripening stage were used in this study. The dates were obtained from Desert Fruit Company (Australia). Date seeds were separated from the fruit and roasted at three intensities (light: 180°C, medium: 200°C, dark: 220°C) in a Qualtex Solidstat oven (Model IW24S, Qualtex Inc., Australia) for 10 min, following the protocol described by Babiker et al. (2020). Roasting temperatures were monitored using the oven's built‐in thermometer, and the roasting level was assessed using the Agtron Scale as a reference. After roasting, the date seeds were ground into powder and sieved through a dual‐layer mesh screen, comprising an 80‐mesh upper layer and a 100‐mesh lower layer. The ground powder (2.5 g) was used to brew date seed coffee‐like beverage using a Breville Creatista® Plus espresso machine under Espresso mode. A consistent pouring volume of 25 mL was maintained at a brewing temperature of 78°C. The abbreviations representing each cultivar and roasting level of the date palm seeds are illustrated in Figure 1.

### 2.3 Phenolic Compounds Estimation

#### 2.3.1 Total Phenolic Content (TPC)

The TPC analysis was conducted using the Folin–Ciocalteu method (Slinkard and Singleton 1977), following the detailed procedure described by Shi et al. (2024). The absorbance was measured in triplicate at 756 nm using a Multiskan GO Microplate Spectrophotometer (Thermo Scientific, Waltham, MA, USA). Gallic acid served as standard, and the TPC data were expressed in mg gallic acid equivalents (GAE)/fresh weight.

#### 2.3.2 Total Flavonoids Content (TFC)

The analysis of TFC was conducted using the AlCl_3_ colorimetric method (Christ and Müller 1960) as described in detail by Shi et al. (2024). The absorbance was measured spectrophotometrically in triplicate at 440 nm. Quercetin was used as standard, and the TFC data were expressed in mg quercetin equivalents (QE)/fresh weight.

#### 2.3.3 Total Condensed Tannin (TCT)

The analysis of TFC was conducted using the vanillin‐sulphuric acid method (Price et al. 1978) as described in detail in the methodology by Shi et al. (2024). The absorbance was measured spectrophotometrically in triplicate at 500 nm. Catechin was used as standard, and the TCT data were expressed in mg catechin equivalents (CE)/fresh weight.

### 2.4 Antioxidant Activities

#### 2.4.1 DPPH Radical Scavenging Capacity Assay (DPPH)

The free radical scavenging activity was determined using a DPPH assay (Blois 1958) as described in detail by Shi et al. (2024). The absorbance was measured spectrophotometrically in triplicate at 517 nm. Trolox was used as standard, and the data were expressed in mg Trolox equivalents (TE)/fresh weight.

#### 2.4.2 Ferric Reducing/Antioxidant Power Assay (FRAP)

The FRAP assay was conducted using the methodology of Sogi et al. (2013), with slight modifications as described by Shi et al. (2024). The absorbance was measured spectrophotometrically in triplicate at 593 nm. A standard curve was created using Trolox, and the data were expressed in mg TE/fresh weight.

#### 2.4.3 Chelating Ability of Ferrous Ion Assay (FICA)

The FICA assay was conducted using the methodology of Dinis et al. (1994) as described in detail by Shi et al. (2024). The absorbance was measured spectrophotometrically in triplicate at 562 nm. Ethylenediaminetetraacetic acid (EDTA) was used for standard curve creation, and the data were expressed in mg EDTA equivalents (EE)/fresh weight.

#### 2.4.4 ABTS Assay

The ABTS activity was determined using the methodology of Re et al. (1999) with slight modifications described by Shi et al. (2024). The absorbance was measured spectrophotometrically in triplicate at 734 nm. A standard curve was created using Trolox, and the data were expressed in mg TE/fresh weight.

#### 2.4.5 Hydroxyl Radical Scavenging Activity Assay (^•^OH‐RSA)

The ^•^OH‐RSA assay was conducted using the methodology of Salgado et al. (2013) as described in detail by Shi et al. (2024). The absorbance was measured spectrophotometrically in triplicate at 510 nm. Trolox was used for standard curve creation and the data were expressed in mg TE/fresh weight.

#### 2.4.6 Reducing Power Assay (RPA)

The RPA assay was conducted using the methodology of Oyaizu (1986) as described by Shi et al. (2024). The absorbance was measured spectrophotometrically in triplicate at 750 nm. Trolox was used for standard curve creation, and the data were expressed in mg TE/fresh weight.

#### 2.4.7 Total Antioxidant Capacity (TAC)

The TAC assay was conducted using the methodology of Prieto et al. (1999) as described by Shi et al. (2024). The absorbance was measured spectrophotometrically in triplicate at 695 nm. A standard curve was created using ascorbic acid. The data were expressed in mg ascorbic acid equivalents (AAE)/fresh weight.

### 2.5 Characterization of Polyphenols

The characterization of polyphenolic compounds in 24 date seed beverage samples was conducted using LC‐ESI‐QTOF‐MS/MS analysis as described before by Shi et al. (2024). An Agilent 1200 Series HPLC system coupled with an Agilent 6530 Accurate‐Mass QTOF LC/MS system equipped with an electrospray ionization (ESI) source was used for the analysis. HPLC mobile phases were sonicated in a 5 L digital ultrasonic water bath (Power Sonic 505, Gyeonggi‐do, Korea) for 10 min at 25°C prior to use. The separation was performed on a Synergi Hydro‐RP 80 Å LC column (250 × 4.6 mm, 4 µm; Phenomenex, Torrance, CA, USA) maintained at 25°C, with the sample compartment held at 10°C. A 20 µL injection volume was used. The binary mobile phase system consisted of solvent A: 100% Milli‐Q water with 0.1% formic acid, and solvent B: acetonitrile/Milli‐Q water/formic acid (95:5:0.1, v/v/v). The flow rate was set to 0.3 mL/min, and the applied gradient program was as follows: 0–2 min hold 2% B, 2–5 min 2%–5% B, 5–25 min 5%–45% B; 25–26 min 45%–100% B, 26–29 min hold 100% B, 29–30 min 100%–2% B, 30–35 min hold 2% B for HPLC equilibration. Both positive and negative modes were applied for peak identification. Nitrogen gas was used as a nebulizer and drying gas (45 psi, 5 L/min at 300°C). Capillary and nozzle voltage were placed at 3.5 and 500 V, respectively. MS/MS analyses were carried out in an automatic mode with collision energy (10, 15, and 30 eV) for fragmentation. Data acquisition and analyses were performed using Agilent LC‐ESI‐QTOF‐MS/MS Mass Hunter workstation software (Qualitative Analysis, version B.03.01, Agilent).

### 2.6 Statistics Analysis

Data analysis was conducted using a one‐way analysis of variance (ANOVA) in Minitab for Windows version 19.0 (Minitab, LLC, State College, PA, USA). Tukey's HSD test was applied for mean separation at a significance level of *p* < 0.05. Pearson's correlation analysis was performed to evaluate the relationships between phenolic content and antioxidant assays. Principal component analysis (PCA) was also used to identify correlations between antioxidant assays and phenolic content across date seed beverage made from eight date cultivars at three roasting levels.

## 3 Results and Discussion

### 3.1 Quantification of Phenolics

The phenolic content of the date seed beverage was assessed using TPC, TFC, and TCT as presented in Table 1. The variations in phenolic composition are illustrated in Supporting Information Figure S1. Overall, the phenolic content in date seed beverage tended to decrease with increasing roasting temperature. Deglet nour cultivar had the highest TPC at light and medium roasting level. However, Bau Strami cultivar had the highest TPC at dark roasting level. Deglet nour, Zahidi, and Bau Strami cultivars had the highest condensed tannin content in light, medium, and dark roasting levels, respectively. Deglet nour cultivar also had the highest TFC in light and medium roasting levels. Seeds from Bau Strami cultivar had the highest TFC in dark roasting level.

**TABLE 1:** Impact of roasting temperatures on phenolics content and antioxidant activities in date seed beverages from different cultivars.

Antioxidant assays	Date cultivar							
	Zahidi	Medjool	Deglet nour	Thoory	Halawi	Barhee	Khadrawy	Bau Strami
Light roasting, 180°C								
	**ZA‐LI**	**ME‐LI**	**DN‐LI**	**TH‐LI**	**HA‐LI**	**BA‐LI**	**KH‐LI**	**BS‐LI**
TPC (mg GAE/g)	8.24 ± 0.47^b*^	4.98 ± 0.16^e^	14.09 ± 0.60^a*^	8.95 ± 0.20^b*^	5.78 ± 0.41^de^	6.94 ± 0.47^c*^	6.33 ± 0.37^cd*^	9.36 ± 0.36^b*^
TCT (mg CE/g)	11.12 ± 0.90^b*^	4.83 ± 0.18^b^	15.65 ± 6.61^a*^	4.68 ± 0.19^b*^	5.68 ± 0.03^b^	8.91 ± 0.33^b*^	8.10 ± 0.43^b*^	11.06 ± 0.32^b*^
TFC (mg QE/g)	0.32 ± 0.02 ^b*^	0.18 ± 0.01^e^	0.57 ± 0.03^a*^	0.35 ± 0.01^b*^	0.21 ± 0.02^de^	0.26 ± 0.02^c*^	0.23 ± 0.02^cd*^	0.36 ± 0.02^b*^
DPPH (mg TE/g)	37.36 ± 0.25^c*^	18.93 ± 1.73^f^	75.95 ± 2.13^a*^	35.97 ± 0.89^cd*^	27.54 ± 0.25^e*^	31.38 ± 0.60^de*^	28.48 ± 0.98^e*^	50.69 ± 3.60^b*^
FRAP (mg TE/g)	16.65 ± 0.01^d*^	10.59 ± 0.36^e^	32.83 ± 2.00^a*^	17.53 ± 0.18^bcd*^	16.91 ± 0.38^cd*^	19.29 ± 0.27^b*^	18.64 ± 0.50^bcd*^	19.03 ± 0.61^bc*^
ABTS (mg AAE/g)	27.08 ± 0.26^b*^	17.03 ± 0.07^d^	52.48 ± 1.26^a*^	24.81 ± 0.06^c*^	24.95 ± 0.18^bc*^	24.87 ± 0.55^bc*^	22.43 ± 1.66^d*^	26.80 ± 0.47^bc*^
^•^OH‐RSA (mg TE/g)	19.46 ± 1.47^c^	29.97 ± 1.74^a^	30.11 ± 3.43^a*^	16.16 ± 1.30^c^	27.41 ± 0.80^ab*^	23.76 ± 2.28^b^	23.61 ± 0.86^b^	18.32 ± 1.00^c^
RPA (mg TE/g)	21.52 ± 0.23^c^	8.28 ± 0.17^e^	53.71 ± 4.40^a*^	26.37 ± 0.71^bc*^	15.63 ± 0.70^d*^	16.59 ± 1.52^d*^	15.63 ± 0.92^d*^	28.83 ± 0.56^b*^
FICA (mg EE/g)	1.95 ± 0.03^d*^	1.69 ± 0.14^e^	2.75 ± 0.05^a*^	2.13 ± 0.11^cd*^	2.35 ± 0.07^bc*^	2.54 ± 0.03^ab*^	2.14 ± 0.07^cd*^	2.34 ± 0.09^bc*^
TAC (mg AAE/g)	0.37 ± 0.01^e^	0.58 ± 0.02^e^	6.98 ± 0.21^a*^	2.19 ± 0.13^c*^	0.06 ± 0.01^f^	0.99 ± 0.10^d^	1.24 ± 0.05^d*^	2.79 ± 0.03^b^
Medium roasting, 200°C								
	**ZA‐ME**	**ME‐ME**	**DN‐ME**	**TH‐ME**	**HA‐ME**	**BA‐ME**	**KH‐ME**	**BS‐ME**
TPC (mg GAE/g)	8.01 ± 0.63^a^	5.75 ± 0.44^b*^	8.65 ± 0.31^a^	5.88 ± 0.41^b^	5.92 ± 0.36^b*^	3.79 ± 0.27^c^	3.66 ± 0.19^c^	7.53 ± 0.45^a^
TCT (mg CE/g)	11.01 ± 0.60^a^	5.16 ± 0.22^d*^	8.06 ± 0.55^bc^	3.21 ± 0.10^e^	7.25 ± 0.49^c*^	3.11 ± 0.17^e^	0.87 ± 0.03^f^	9.06 ± 0.55^b^
TFC (mg QE/g)	0.31 ± 0.03^a^	0.21 ± 0.02^b^	0.33 ± 0.01^a^	0.21 ± 0.02^b^	0.22 ± 0.02^b*^	0.12 ± 0.01^c^	0.12 ± 0.01^c^	0.29 ± 0.02^a^
DPPH (mg TE/g)	36.57 ± 0.73^a^	22.21 ± 1.45^de*^	26.64 ± 1.57^c^	23.18 ± 1.15^d^	19.08 ± 0.74^e^	15.21 ± 1.39^f^	9.31 ± 0.42^g^	30.46 ± 1.18^b^
FRAP (mg TE/g)	15.97 ± 0.55^b^	13.4 ± 0.33^c*^	17.77 ± 0.39^a^	15.21 ± 0.43^b^	12.36 ± 0.33^d^	7.10 ± 0.16^e^	6.41 ± 0.11^e^	17.62 ± 0.41^a^
ABTS (mg AAE/g)	23.17 ± 0.71^bc^	20.95 ± 0.14^cd*^	27.74 ± 0.70^a^	21.41 ± 0.61^bcd^	20.81 ± 0.16^d^	15.81 ± 0.20^e^	12.90 ± 0.39^f^	23.37 ± 1.87^b^
^•^OH‐RSA (mg TE/g)	16.39 ± 0.50^d^	24.67 ± 1.68^ab^	20.77 ± 0.93^bc^	28.21 ± 1.79^a*^	20.69 ± 0.08^c^	26.49 ± 0.32^a*^	26.27 ± 2.49^a*^	21.52 ± 1.41^bc^
RPA (mg TE/g)	23.85 ± 1.48^a*^	12.31 ± 0.58^c*^	26.44 ± 2.16^a^	12.67 ± 0.93^c^	10.45 ± 0.36^c^	2.67 ± 0.23^d^	2.38 ± 0.17^d^	17.12 ± 0.21^b^
FICA (mg EE/g)	1.67 ± 0.04^d^	1.26 ± 0.07^e*^	2.09 ± 0.10^b^	1.98 ± 0.04^bc^	1.72 ± 0.04^d^	2.44 ± 0.08^a^	1.85 ± 0.10^c^	1.98 ± 0.07^bc^
TAC (mg AAE/g)	2.43 ± 0.22^b*^	0.17 ± 0.01^f*^	3.45 ± 0.13^a^	1.03 ± 0.03^de^	0.44 ± 0.03^f*^	1.15 ± 0.05^d*^	0.76 ± 0.03^e^	1.86 ± 0.14^c^
Dark roasting, 220°C								
	**ZA‐DA**	**ME‐DA**	**DN‐DA**	**TH‐DA**	**HA‐DA**	**BA‐DE**	**KH‐DA**	**BS‐DA**
TPC (mg GAE/g)	4.18 ± 0.08^b^	4.11 ± 0.28^b^	3.20 ± 0.05^c^	1.66 ± 0.06^e^	1.82 ± 0.02^e^	1.82 ± 0.05^e^	2.39 ± 0.04^d^	6.33 ± 0.38^a^
TCT (mg CE/g)	1.56 ± 0.02^b^	0.92 ± 0.07^c^	0.88 ± 0.01^c^	ND	ND	0.46 ± 0.04^d^	ND	3.15 ± 0.05^a^
TFC (mg QE/g)	0.14 ± 0.01^b^	0.25 ± 0.02^a*^	0.10 ± 0.01^c^	0.03 ± 0.01^d^	0.05 ± 0.01^d^	0.04 ± 0.01^d^	0.21 ± 0.02^a^	0.23 ± 0.02^a^
DPPH (mg TE/g)	14.78 ± 0.36^a^	14.49 ± 0.60^a^	9.81 ± 0.22^c^	6.99 ± 0.10^d^	5.83 ± 0.26^de^	4.76 ± 0.23^e^	11.60 ± 0.36^b^	15.62 ± 1.22^a^
FRAP (mg TE/g)	9.32 ± 0.19^b^	6.34 ± 0.58^c^	6.62 ± 0.36^c^	3.40 ± 0.13^e^	2.68 ± 0.12^f^	3.37 ± 0.08^e^	5.10 ± 0.21^d^	11.29 ± 0.42^a^
ABTS (mg AAE/g)	10.24 ± 0.41^b^	8.98 ± 0.84^c^	10.95 ± 0.43^ab^	6.94 ± 0.19^d^	3.52 ± 0.10^e^	6.07 ± 0.41^d^	6.90 ± 0.05^d^	11.61 ± 0.30^a^
^•^OH‐RSA (mg TE/g)	24.40 ± 2.11^a*^	27.96 ± 2.17^a*^	19.47 ± 1.56^b^	5.42 ± 0.48^d^	13.67 ± 0.48^c^	19.44 ± 1.38^b^	22.59 ± 0.87^ab^	28.27 ± 1.17^a*^
RPA (mg TE/g)	9.78 ± 0.26^b^	8.49 ± 0.13^b^	6.18 ± 0.42^c^	1.42 ± 0.07^d^	1.94 ± 0.09^d^	0.37 ± 0.03^e^	6.17 ± 0.49^c^	15.64 ± 0.71^a^
FICA (mg EE/g)	0.60 ± 0.02^b^	0.16 ± 0.01^c^	0.88 ± 0.04^a^	0.70 ± 0.05^b^	0.10 ± 0.01^c^	0.87 ± 0.03^a^	0.10 ± 0.01^c^	0.91 ± 0.05^a^
TAC (mg AAE/g)	0.95 ± 0.09^b^	0.03 ± 0.01^d^	0.24 ± 0.02^c^	0.84 ± 0.03^b^	ND	0.99 ± 0.01^b^	ND	3.23 ± 0.16^a*^

*Note*: The data are shown as mean ± standard deviation (*n* = 3); ^a–e^ indicate the means in a row with significant difference (*p* < 0.05) using one‐way ANOVA and Tukey's test; ^*^ indicate the highest value among the three roasting levels; ND = not detected. The standards and samples are mentioned in abbreviations. AAE, ascorbic acid equivalents; CE, catechin equivalents; EE, EDTA equivalents; GAE, gallic acid equivalents; QE, quercetin equivalents; TE, Trolox equivalents.

**TABLE 2:** Pearson's correlation between phenolic content and antioxidant assays.

	TPC	TCT	TFC	DPPH	FRAP	ABTS	^•^OH‐RSA	RPA	FICA
TCT	0.904**								
TFC	0.957**	0.837**							
DPPH	0.951**	0.906**	0.927**						
FRAP	0.956**	0.909**	0.900**	0.940**					
ABTS	0.944**	0.907**	0.885**	0.941**	0.966**				
^•^OH‐RSA	0.248*	0.132	0.294*	0.194*	0.303*	0.294*			
RPA	0.969**	0.853**	0.953**	0.958**	0.928**	0.916**	0.183		
FICA	0.715**	0.752**	0.577**	0.693**	0.775**	0.812**	0.277	0.597	
TAC	0.786**	0.637**	0.734**	0.749**	0.711**	0.717**	0.158	0.838**	0.472*

^**^ Significant correlation at *p ≤* 0.01; ^*^ significant correlation at *p ≤* 0.05.

Deglet nour had the highest TPC (14.09 mg GAE/g) across all the lightly roasted date seed beverages. This cultivar maintained the highest TPC at the medium roasting level (8.65 mg GAE/g), representing a 38.6% reduction from light roasting. However, a substantial decline was observed at the dark roasting level, where TPC decreased to 3.20 mg GAE/g, representing a 77.3% reduction from light to dark roasting level. In contrast, Bau Strami cultivar maintained the highest TPC value at dark roasting level (6.33 mg GAE/g), indicating a superior thermal stability, with a lower reduction percentage, compared to Deglet nour (from 9.36 to 6.33 mg GAE/g; 32.4% reduction). Despite the inherent differences in TPC among the various date seed cultivars, we observed a consistent trend of decreasing TPC with increasing roasting temperature. This phenomenon is consistent with previous research, demonstrating that high temperature can potentially induce the degradation of phenolic compounds, resulting in a decrease in TPC (Maghsoudlou et al. 2019). The reductions observed in this study were consistent with earlier research but exceeded the phenolic losses reported in roasted Arabica and Robusta coffee beans, ranging from 8.68% to 22.4% (Anh‐Dao et al. 2024).

A comparative analysis demonstrated that the TPC of date seed beverages, even at the light roasting level (14.09 mg GAE/g), was lower than that of green tea (125.7–178.4 mg GAE/g) but comparable to coffee beans roasted from green to dark (14.92–16.55 mg GAE/g; Alnsour et al. 2022; Meyer et al. 2023). Particularly, the date seed beverage performed better than certain agricultural byproducts, such as water‐extracted almond skin (1.32–2.25 mg GAE/g), positioning date seed as a potential phenolic‐rich source among waste‐derived materials (Timón et al. 2022). However, the substantial thermal degradation observed in date seeds (e.g., 77.3% TPC loss in Deglet nour) contrasts with milder losses in coffee beans (8.68%–22.4%) from light roasted (195°C–210°C) to dark roasted (230°C and 240°C; Anh‐Dao et al. 2024), highlighting the need for optimized roasting protocols for minimal degradation of phenolic compounds in date seed beverages.

Among the light roasted date seed beverages, Deglet nour cultivar had the highest TCT (15.65 mg CE/g), but at the medium roasting level, Zahidi cultivar showed the highest TCT (11.01 mg CE/g). Bau Strami cultivar had the highest TCT after dark roasting level (3.15 mg CE/g). Overall, TCT content generally decreased as the roasting temperature increased, mirroring the trend observed for TPC. However, the decline in TCT was more significant relative to the reduction observed in TPC. For example, Deglet nour cultivar experienced a 94.4% loss in TCT, declining from 15.65 mg CE/g at the light roast to 0.88 mg CE/g at the dark roast. In addition, TCT concentration in the Thoory, Halawi, and Khadrawy cultivars became undetectable after dark roasting, highlighting the high thermal sensitivity of these compounds.

The decrease in TCT content with increasing roasting temperature was previously reported by Jylhä et al. (2021), as they observed a significant thermal degradation of condensed tannins in Norway spruce bark. The TCT content in date seeds beverage (ranging from 0.46 to 15.65 mg CE/g) exceeded that reported in other waste‐derived materials, such as stone fruit waste (0.07–0.19 mg CE/g; Hong et al. 2021). Despite the reduction in TCT and a loss of nutritional value, the development of date seed coffee as a beverage requires considering consumer preference, and future studies should incorporate sensory evaluation to explore the optimal balance between nutritional value and consumer acceptance.

In the TFC assay, the Deglet nour cultivar consistently had the highest TFC values in both lightly (0.57 mg QE/g) and medium‐roasted (0.33 mg QE/g) samples. However, Medjool date seed beverages demonstrated the highest TFC (0.25 mg QE/g) at the dark roasting level. The overall trend mirrored the decline observed in TPC and TCT, with TFC generally decreasing with increasing roasting intensity. However, a significant exception was observed in Medjool date seed beverages. In this cultivar, TFC values exhibited an upward trend with increasing roasting intensity (light: 0.18 mg QE/g, medium: 0.21 mg QE/g, dark: 0.25 mg QE/g). This observation may suggest that the roasting process enhances the release of flavonoids from Medjool date seeds into the brewing water. However, further investigation is necessary to confirm this hypothetical explanation. This unique characteristic of Medjool date seeds could offer a significant advantage for the valorization of date seed waste, particularly for producing beverages with enhanced flavonoid content. However, TFC in Medjool date seed beverage (0.25 mg QE/g) was substantially lower than that reported for cocoa pod husk peel extracts (1.96–4.34 mg QE/g). This may suggest that date seed beverage may not be a rich source of flavonoids, compared to other food waste‐derived products and highlights the need for further improvement through optimization of roasting and brewing techniques (Yahya et al. 2021).

### 3.2 Assessment of Antioxidant Activity

The antioxidant capacity of beverages prepared from each roasting level of eight date seed cultivars was assessed using different assays (Table 1). The variations in antioxidant capacity are shown in Supporting Information Figure S1. These assays evaluated the capacity of the beverages to neutralize free radicals and reactive oxygen species, which are known to induce oxidative stress, damaging biological molecules and playing a role in numerous health‐related problems (Li et al. 2016).

Generally, the antioxidant capacity of date seed beverages decreased with increasing roasting temperature, which mirrored the decline observed in TPC (Supporting Information Figure S1). From a cultivar perspective, the Deglet nour cultivar consistently demonstrated the highest antioxidant activity (across lightly and medium‐roasted samples) in six and four antioxidant assays. However, the Bau Strami cultivar had the highest values in all seven antioxidant assays after dark roasting. This may suggest that the optimal roasting level and cultivar selection significantly influence the antioxidant properties of the final beverage.

At the light roasting level, Deglet nour had higher values in all seven antioxidant assays, compared to other cultivars. In contrast, the Medjool cultivar displayed the lowest results in five assays. Antioxidant activity generally decreased across all assays after medium roasting, compared to light roasting. Deglet nour cultivar had the highest values in four assays (FRAP, ABTS, RAP, and TAC) at this level.

A further decline in antioxidant activity was observed after dark roasting, compared to both light and medium roasting levels. For example, DPPH radical scavenging activity in beverage made from Deglet nour cultivar decreased by 87.1%, from 75.95 mg TE/g at light roast to 9.81 mg TE/g at dark roast. In contrast, the Bau Strami cultivar demonstrated greater antioxidant retention, maintaining 69.2% of its DPPH activity (from 50.69 to 15.62 mg TE/g) and 59.3% of its FRAP activity (from 19.03 to 11.29 mg TE/g) across the same roasting intensity. Zahidi, showing the highest DDPH value at medium roasting (36.57 mg TE/g), demonstrated a substantial decrease to 14.78 mg TE/g at dark roasting, which is consistent with the expected degradation of phenolic compounds at higher temperatures (Maghsoudlou et al. 2019). However, an unexpected trend was observed in ^•^OH‐RSA values. Although a general decrease was expected, some cultivars exhibited an increase in ^•^OH‐RSA activity after a higher temperature roasting. This could potentially be ascribed to the generation of novel antioxidant compounds during roasting or cultivar‐specific variations and thus requires further research for a deeper understanding of the mechanism involved.

It is important to recognize that plant seeds have shown substantial variability in their responses to roasting. For example, in a study by Peng et al. (2021), pumpkin seeds demonstrated an increasing trend in phenolic content during roasting, with values rising from 2.44 to 3.82 mg GAE/g as the temperature increased to 200°C. This was accompanied by an increase in DPPH radical scavenging activity, from 0.31 to 0.47 µmol TE/g. In contrast, sacha inchi seeds displayed a markedly different response. In the study by Kittibunchakul et al. (2023), the TPC of raw sacha inchi seeds (45.2 mg GAE/100 g) decreased to 40.8 mg GAE/100 g after hot‐air tray dryer roasting. However, despite this reduction in phenolic content, both FRAP values (from 2.47 to 4.27 µmol TE/100 g) and DPPH activity (from 0.012 to 0.018 µmol TE/100 g) increased, likely suggesting the formation of new antioxidant‐active compounds during the roasting process.

### 3.3 Statistical Relationship Evaluation

**FIGURE 2:**
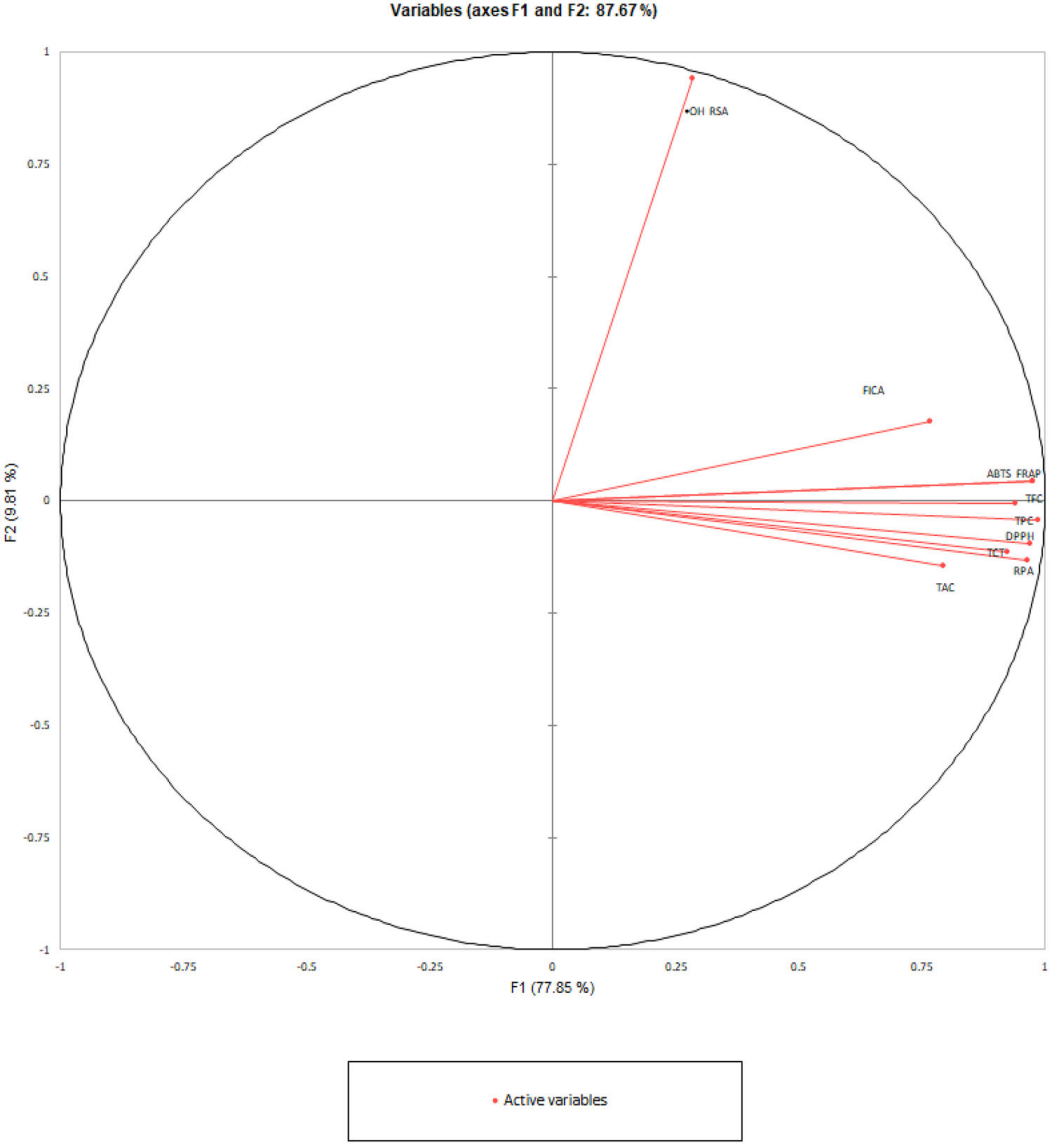
Principal component analysis of phenolic content (TPC, TFC, and TCT) and antioxidant activities (FICA, ^•^OH‐RSA, RPA, DPPH, ABTS, FRAP and TAC) from date seed beverages made from eight date fruit cultivars at three roasting levels.

Pearson's correlation analysis showed significant correlations between estimated phenolic content and most of the antioxidant assays (Table 2). However, a weaker correlation was observed between these parameters and ^•^OH‐RSA (^•^OH‐RSA and TPC: *r* = 0.248; ^•^OH‐RSA and TCT: *r* = 0.132; ^•^OH‐RSA and TFC: *r* = 0.294). This observation was further supported by the PCA graph (Figure 2), where ^•^OH‐RSA showed a distinct separation from the cluster of phenolic compounds and other antioxidant assays. In support of this finding, Shi et al. (2024) suggested that ^•^OH‐RSA activity in date seeds might be influenced by other antioxidant compounds beyond the measured phenolics.

**TABLE 3:** Characterization of polyphenols in date seed beverages by LC‐ESI‐QTOF‐MS/MS.

No.	Proposed compounds	Molecular formula	RT (min)	Ionization (ESI+/ESI−)	Molecular Weight	Theoretical (*m*/*z*)	Observed (*m*/*z*)	Error (ppm)	MS/MS product ion	Sample
		**Phenolic acids** **hydroxybenzoic acids**								
1	Ellagic acid acetyl‐arabinoside	C_21_H_16_O_13_	4.076	[M − H]^−^	476.0584	475.0511	475.0501	−2.1	301	ME‐DA
2	Ellagic acid glucoside	C_20_H_16_O_13_	4.158	[M − H]^−^	464.0586	463.0513	463.0515	0.4	301	*KH‐DA, HA‐DA, ME‐DA
3	Gallic acid 4‐*O*‐glucoside	C_13_H_16_O_10_	14.257	**[M − H]^−^	332.0735	331.0662	331.0665	0.9	331	*ME‐DA, TH‐ME, HA‐DA, KH‐DA
4	4‐*O*‐Methylgallic acid	C_8_H_8_O_5_	16.155	[M + H]^+^	184.0357	185.0430	185.0423	−3.8	170, 142	TH‐DA
5	Ellagic acid arabinoside	C_19_H_14_O_12_	25.114	[M − H]^−^	434.0463	433.0390	433.0391	0.2	300	*HA‐DA, KH‐DA, ME‐DA
6	Ellagic acid	C_14_H_6_O_8_	27.334	[M − H]^−^	302.0064	300.9991	300.9990	−0.3	284, 271, 257, 229	ME‐DA
		**Hydroxycinnamic acids**								
7	Caffeic acid 3‐sulfate	C_33_H_40_O_18_	8.784	[M − H]^−^	259.9971	258.9898	258.9898	0.8	215, 135	*KH‐DA, HA‐DA, ME‐DA
8	5‐Feruloylquinic acid	C_25_H_24_O_12_	12.718	**[M − H]^−^	368.1093	367.1020	367.1020	−1.9	192, 193	*HA‐LI, ZA‐LI, ZA‐ME, BS‐ME, BS‐DA, BA‐LI, BA‐ME, TH‐LI, TH‐ME, TH‐DA, DE‐LI, HA‐ME, KH‐LI, KH‐ME, ME‐LI, ME‐ME, ME‐DA
9	*p*‐Coumaroyl tartaric acid	C_20_H_18_O_8_	25.268	**[M − H]^−^	296.0533	295.0460	295.0460	0.7	115	*KH‐DA, DE‐ME, HA‐DA, ME‐DA
10	*p*‐Coumaroyl malic acid	C_17_H_20_O_9_	28.511	**[M − H]^−^	280.0582	279.0509	279.0509	0.7	163, 119	*HA‐DA, ZA‐LI, KH‐DA, ME‐DA
11	Caffeic acid	C_9_H_8_O_4_	29.623	**[M − H]^−^	180.0407	179.0334	179.0334	1.1	143, 133	*KH‐DA, TH‐LI
12	5‐*p*‐Coumaroylquinic acid	C_16_H_18_O_8_	38.144	[M − H]^−^	338.1001	337.0928	337.0928	−0.9	265, 173, 162	HA‐DA
13	Caffeoyl glucose	C_15_H_18_O_9_	42.081	**[M − H]^−^	342.0934	341.0861	341.0861	−1.5	179, 161	*BS‐LI, BA‐ME, TH‐LI, DE‐LI, ME‐LI, HA‐DA
14	3‐Caffeoylquinic acid	C_16_H_18_O_9_	42.523	**[M − H]^−^	354.0916	353.0843	353.0843	−0.3	253, 190, 144	*ME‐LI, ZA‐LI, ZA‐DA, BS‐LI, TH‐LI, HA‐LI
		**Hydroxyphenylacetic acids**								
15	3,4‐Dihydroxyphenylacetic acid	C_8_H_8_O_4_	38.688	[M − H]^−^	168.0421	167.0348	167.0347	−0.6	149, 123	*ME‐ME, ZA‐ME, ZA‐DA, BA‐DA, DE‐ME, KH‐DA, ME‐DA
		**Hydroxyphenylpentanoic acids**								
16	Dihydroferulic acid 4‐sulfate	C_10_H_12_O_7S_S	22.394	**[M − H]^−^	276.0276	275.0203	275.0205	0.7	195, 151, 177	*KH‐DA, ZA‐ME, BA‐DA, TH‐ME
17	5‐(3',4',5'‐trihydroxyphenyl)‐γ‐valerolactone	C_12_H_14_O4	64.473	[M + H]^+^	222.0899	223.0972	223.0973	0.4	205, 163	ZA‐LI
		**Flavonoids** **Anthocyanins**								
18	Delphinidin 3‐*O*‐(6''‐acetyl‐galactoside)	C_23_H_23_O_13_	56.351	[M + H]^+^	507.1183	508.1256	508.1232	−4.7	303, 507	BS‐DA
19	Petunidin 3‐*O*‐(6''‐acetyl‐glucoside)	C_24_H_25_O_13_	57.823	[M + H]^+^	521.1322	522.1395	522.1389	−1.1	317	KH‐LI
20	Peonidin 3‐*O*‐diglucoside‐5‐*O*‐glucoside	C_34_H_43_O_21_	59.803	**[M + H]^+^	787.2302	788.2375	788.2381	0.8	610, 464	*ZA‐LI, BS‐ME
21	Pelargonidin 3‐*O*‐rutinoside	C_27_H_31_O_14_	60.450	[M + H]^+^	579.1680	580.1753	580.1740	−2.2	271, 433	KH‐LI
22	Cyanidin 3,5‐*O*‐diglucoside	C_27_H_31_O_16_	61.155	[M + H]^+^	611.1582	612.1655	612.1662	1.1	449, 287	*ME‐LI, TH‐LI
23	Cyanidin 3‐*O*‐(6''‐*p*‐coumaroyl‐glucoside)	C_30_H_27_O_13_	61.388	[M + H]^+^	595.1429	596.1502	596.1511	1.5	287	*BS‐DA, ME‐LI
24	Cyanidin 3‐*O*‐(6''‐malonyl‐3''‐glucosyl‐glucoside)	C_30_H_33_O_19_	61.836	**[M + H]^+^	697.1630	698.1703	698.1686	−2.4	449, 180, 88	*HA‐ME, HA‐DA
25	Delphinidin 3‐*O*‐glucosyl‐glucoside	C_27_H_31_O_17_	65.038	[M + H]^+^	627.1560	628.1633	628.1633	0.0	465, 303	DE‐ME
		**Flavanols**								
26	Cinnamtannin A2	C_60_H_50_O_24_	4.156	[M − H]^−^	1154.2709	1153.2636	1153.2601	−3.0	739	HA‐DA
27	4'‐*O*‐Methylepigallocatechin	C_16_H_16_O_7_	15.539	[M + H]^+^	320.0874	321.0947	321.0953	1.9	92, 121	*BA‐LI, ME‐DA
28	3'‐*O*‐Methylcatechin	C_16_H_16_O_6_	26.259	**[M − H]^−^	304.0945	303.0872	303.0876	1.3	271, 163	*KH‐DA, TH‐ME, ME‐ME, HA‐DA, ME‐DA
29	(+)‐Gallocatechin	C_15_H_14_O_7_	34.129	**[M − H]^−^	306.0729	305.0656	305.0650	−2.0	261, 219	*ME‐DA, HA‐ME, BA‐ME
30	Procyanidin dimer B7	C_30_H_26_O_12_	40.846	**[M − H]^−^	578.1391	577.1318	577.1318	0.0	451	*ZA‐LI, ZA‐ME, BS‐LI, BS‐ME, BA‐LI, BA‐DA, TH‐LI, DE‐LI, DE‐DA, HA‐ME, KH‐LI, KH‐ME, ME‐LI, ME‐ME
31	Procyanidin trimer C1	C_45_H_38_O_18_	41.127	**[M − H]^−^	866.1993	865.1920	865.1928	0.9	739, 713, 695	*ZA‐LI, ZA‐ME, BS‐LI, BS‐ME, TH‐ME, DE‐LI, DE‐ME, HA‐LI
32	(‐)‐Epicatechin	C_15_H_14_O_6_	44.048	**[M − H]^−^	290.0767	289.0694	289.0695	0.3	273, 139, 291	*TH‐ME, ZA‐LI, ZA‐ME, ZA‐DA, BS‐LI, BS‐ME, TH‐DA, DE‐LI, DE‐ME, DE‐DA, HA‐LI, KH‐LI, ME‐DA
		**Flavanones**								
33	Narirutin	C_27_H_32_O_14_	13.447	**[M − H]^−^	580.1797	579.1724	579.1726	0.3	271	*HA‐ME, DE‐ME, KH‐LI
34	Naringin 4'‐*O*‐glucoside	C_33_H_42_O_19_	57.225	**[M − H]^−^	742.2316	741.2243	741.2244	0.1	433, 271	*HA‐DA, BA‐LI, ME‐DA
35	8‐Prenylnaringenin	C_20_H_20_O_5_	57.904	[M + H]^+^	340.1321	341.1394	341.1402	2.3	323, 271, 137	BS‐ME
36	Neohesperidin	C_28_H_34_O_15_	60.821	**[M + H]^+^	610.1873	611.1946	611.1934	−2.0	593, 465, 449, 303	*BS‐DA, TH‐LI, HA‐ME, ME‐LI, ME‐ME
		**Flavones**								
37	Apigenin 6,8‐di‐*C*‐glucoside	C_27_H_30_O_15_	42.67	**[M − H]^−^	594.1530	593.1457	593.1451	−1.0	503, 473	*DE‐LI, ZA‐DA
38	Apigenin 7‐*O*‐(6''‐malonyl‐apiosyl‐glucoside)	C_29_H_30_O_17_	46.476	[M − H]^−^	650.1551	649.1378	649.1379	0.2	605	*KH‐DA, HA‐DA
39	Cirsilineol	C_18_H_16_O_7_	55.665	**[M + H]^+^	344.0899	345.0972	345.0966	−1.7	330, 312, 297, 284	*BS‐ME, HA‐DA, KH‐DA, ME‐DA
40	Scutellarein	C_15_H_10_O_6_	57.234	[M + H]^+^	286.0491	287.0564	287.0572	2.8	269, 259	*ZA‐DA
41	Luteolin 7‐*O*‐(2‐apiosyl‐glucoside)	C_26_H_28_O_15_	61.782	**[M + H]^+^	580.1454	581.1527	581.1519	−1.4	180, 324	*KH‐LI, BS‐ME, TH‐LI
42	Chrysoeriol 7‐*O*‐(6''‐malonyl‐apiosyl‐glucoside)	C_30_H_32_O_18_	64.171	[M + H]^+^	680.1583	681.1656	681.1681	3.7	177, 339, 179 341	ME‐LI
43	Neodiosmin	C_28_H_32_O_15_	66.173	[M + H]^+^	608.1711	609.1784	609.1787	0.5	301, 286	*HA‐ME, ZA‐LI, ZA‐ME
44	Nobiletin	C_21_H_22_O_8_	66.655	**[M + H]^+^	402.1313	403.1386	403.1380	−1.5	388, 373	*DE‐LI, BS‐ME, HA‐LI
45	Apigenin 7‐*O*‐glucuronide	C_21_H_18_O_11_	68.527	[M + H]^+^	446.0878	447.0951	447.0965	3.1	271, 253	*ME‐DA, HA‐DA
		**Flavonols**								
46	Quercetin 3'‐*O*‐glucuronide	C_21_H_18_O_13_	4.076	[M − H]^−^	478.0742	477.0669	477.0678	1.9	301	*ME‐DA, HA‐DA
47	Quercetin 3'‐sulfate	C_15_H_10_O_10_S	4.085	**[M − H]^−^	382.0010	380.9937	380.9938	0.3	79	*KH‐DA, BS‐ME
48	Quercetin 3‐*O*‐rutinoside	C_27_H_30_O_16_	49.696	*[M − H]^−^	610.1517	609.1444	609.1444	0.0	447, 463, 301	*HA‐DA, TH‐ME, KH‐DA, ME‐DA
49	Quercetin 3‐*O*‐glucosyl‐xyloside	C_26_H_28_O_16_	52.132	**[M − H]^−^	596.1350	595.1277	595.1271	−1.0	265, 138, 115, 144	*KH‐DA, ME‐DA, BA‐DA
50	Quercetin 3‐*O*‐xylosyl‐glucuronide	C_26_H_26_O_17_	61.354	[M + H]^+^	610.1187	611.1260	611.1290	4.9	479, 303, 285, 239	BA‐ME
51	Quercetin 3‐*O*‐(6"‐malonyl‐glucoside) 7‐*O*‐glucoside	C_30_H_32_O_20_	65.036	[M + H]^+^	712.1469	713.1542	713.1550	1.1	445, 300, 169	ME‐LI
		**Isoflavonoids**								
52	6''‐*O*‐Malonylglycitin	C_25_H_24_O_13_	12.510	[M + H]^+^	532.1242	533.1315	533.1308	−1.3	285, 270, 253	*BA‐LI, ZA‐ME
53	6''‐*O*‐Acetylglycitin	C_24_H_24_O_11_	12.966	[M + H]^+^	488.1289	489.1362	489.1358	−0.8	285, 270	*BS‐ME, BS‐LI, BA‐LI
54	3'‐Hydroxydaidzein	C_15_H_10_O_5_	16.446	[M + H]^+^	270.0531	271.0604	271.0606	0.7	253,241, 225	*TH‐DA, BS‐LI, BA‐ME, TH‐ME
55	5,6,7,3',4'‐Pentahydroxyisoflavone	C_15_H_10_O_7_	28.458	[M + H]^+^	302.0427	303.0500	303.0502	0.7	285, 257	BA‐ME
56	6''‐*O*‐Malonyldaidzin	C_24_H_22_O_12_	59.131	[M + H]^+^	502.1086	503.1159	503.1164	1.0	255	TH‐LI
57	6''‐*O*‐Malonylgenistin	C_24_H_22_O_13_	68.817	[M + H]^+^	518.1053	519.1126	519.1129	0.6	271	ME‐DA
		**Other polyphenols** **Alkylmethoxyphenols**								
58	4‐Vinylsyringol	C_15_H_14_O_3_	63.386	[M + H]^+^	242.0944	243.1017	243.1014	−1.2	225, 211, 197	*DE‐DA, HA‐LI
		**Furanocoumarins**								
59	Isopimpinellin	C_13_H_10_O_5_	58.122	[M + H]^+^	246.053	247.0603	247.0606	1.2	232, 217, 205, 203	DE‐LI
		**Hydroxybenzoketones**								
60	2‐Hydroxy‐4‐methoxyacetophenone 5‐sulfate	C_9_H_10_O_7_S	4.122	**[M − H]^−^	262.0129	261.0056	261.0059	1.1	181, 97	*KH‐DA, ZA‐DA, BA‐DA, HA‐DA, ME‐DA,
		**Hydroxycoumarins**								
61	Scopoletin	C_10_H_8_O_4_	23.882	[M − H]^−^	192.0435	191.0362	191.0361	−0.5	176	*BA‐LI, HA‐ME, KH‐ME
62	Coumarin	C_9_H_6_O_2_	65.675	[M + H]^+^	146.0363	147.0436	147.0435	−0.7	103, 91	*DE‐DA, ZA‐LI, BS‐ME, BA‐ME, TH‐ME, TH‐DA, HA‐ME
		**Hydroxybenzaldehydes**								
63	*p*‐Anisaldehyde	C_8_H_8_O_2_	31.126	[M + H]^+^	136.0520	137.0593	137.0589	−2.9	122, 109	TH‐DA
		**Hydroxyphenylpropenes**								
64	Eugenol	C_10_H_12_O_2_	62.921	[M + H]^+^	164.0842	165.0925	165.0915	0.0	31	TH‐ME
		**Lignans**								
65	Schisandrin	C_24_H_32_O_7_	12.011	[M + H]^+^	432.2160	433.2233	433.2231	−0.5	415, 384, 361	*BS‐ME, ZA‐ME, ME‐ME
66	7‐Oxomatairesinol	C_20_H_20_O_7_	62.291	**[M + H]^+^	372.1227	373.1300	373.1288	−3.2	358, 343, 328, 325	*DE‐DA, KH‐DA
67	Schisandrol B	C_23_H_28_O_7_	62.467	[M + H]^+^	416.1851	417.1924	417.1935	2.6	224, 193, 165	*BS‐LI, BA‐ME
68	Schisandrin B	C_23_H_28_O_6_	63.359	[M + H]^+^	400.1869	401.1942	401.1948	1.5	386	*HA‐ME, BS‐ME, ME‐ME
69	Schisandrin C	C_22_H_24_O_6_	63.392	**[M + H]^+^	384.1588	385.1661	385.1651	−2.6	370, 315, 300	*TH‐DA, TH‐ME, KH‐DA

*Note*: *Compound was detected in more than one sample, data presented in this table are from asterisk sample. **Compounds were detected in both negative [M − H]^−^ and positive [M+H]^+^ mode of ionization while only single mode data was presented. Date coffee samples are mentioned in abbreviations.

Overall, despite the exception observed in •OH‐RSA, a positive strong relationship was identified between phenolic content and antioxidant activity. Therefore, to maximize antioxidant activity in date seed beverages, careful control of the roasting temperature is crucial to minimize the thermal degradation and inactivation of phenolic compounds.

### 3.4 Characterization of Polyphenols

The polyphenolic compounds in date seed beverages were qualitatively analyzed using LC‐ESI‐QTOF‐MS/MS in both negative and positive ionization modes (Table 3). Phenolic compounds in this table were tentatively identified using theoretical *m/z* values, isotope distributions, and MS/MS spectra, analyzed with Agilent MassHunter software and the Personal Compound Database and Library. Compounds with a mass error of less than ±5 ppm were selected for further MS/MS identification and structural characterization. This analysis identified 69 phenolic compounds, consisting of 17 phenolic acids, 40 flavonoids, and 12 additional polyphenols.

#### 3.4.1 Phenolic Acids

There were 17 phenolic acids detected, comprising seven hydroxycinnamic acids, five hydroxybenzoic acids, one hydroxyphenylacetic acid, and two hydroxyphenylpentanoic acids.

##### 3.4.1.1 Hydroxybenzoic acid

Six hydroxybenzoic acids were identified, with two of these compounds tentatively identified as ellagic acid acetyl‐arabinoside (Compound 1) and ellagic acid (Compound 6) and were exclusively detected in dark‐roasted Medjool date seed beverages (ME‐DA). Compound 2 was preliminarily identified as ellagic acid glucoside ([M − H]− *m*/*z* 463.0515) and was also unique to dark‐roasted samples. This compound, previously reported in date seeds (Shi et al. 2023) and mango peel (Peng et al. 2019), was verified by the presence of a production at *m/z* 301.

Gallic acid 4‐*O*‐glucoside (Compound 3, RT = 14.257 min, *m/z* 331.0665), detected in four samples, was the most prevalent hydroxybenzoic acid among all samples. This compound, also reported in various fruit peels (Suleria et al. 2020), exhibited a product ion at *m/z* 331.

4‐*O*‐Methylgallic acid (Compound 4, [M − H]^−^ *m/z* 185.0423), detected only in negative mode verified by product ions at *m/z* 170 and 142, was uniquely found in dark‐roasted Thoory date seed beverages. Dragon fruit peel has previously been reported to contain this compound (Liu et al. 2024).

##### 3.4.1.2 Hydroxycinnamic acids

Hydroxycinnamic acids was the main type of phenolic acids in date seed beverages, with eight compounds identified in this study. 5‐Feruloylquinic acid (Compound 8), detected in both modes (*m/z* 367.1020, product ions at *m/z* 192 and 193), was widely distributed across samples. As reported by Shi et al. (2024), the presence of this compound in mature (*Tamar*) stage date seeds suggests its stability at high temperatures. Red seaweed has also been reported to contain 5‐feruloylquinic acid (Ebrahimi et al. 2024).

Caffeic acid (Compound 11), a well‐known antioxidant compound (RT = 29.623 min, *m/z* 179.0334) was detected in dark‐roasted Khadrawy and light‐roasted Thoory samples. Product ions at *m/z* 143 and 133 confirmed the identity of this compound (Lin et al. 2019).

Caffeoyl glucose (Compound 13; RT = 42.081 min, *m/z* 341.0861) and 3‐caffeoylquinic acid (Compound 14; RT = 42.523 min, *m/z* 353.0843) were primarily observed in lightly roasted date seed beverages and were absent in medium‐ and dark‐roasted samples. This suggests their susceptibility to thermal degradation, aligning with previous studies demonstrating the heat‐sensitivity of certain phenolic compounds (Maghsoudlou et al. 2019).

##### 3.4.1.3 Hydroxyphenylacetic acids

Compound 15 was preliminarily characterized as 3,4‐dihydroxyphenylacetic acid in only negative mode with the precursor ion observed at *m/z* 167.0347. This compound was exclusively detected in medium‐ and dark‐roasted date seed beverage samples. This may suggest that high temperatures not only degrade phenolic compounds but may also facilitate their release from the date seed matrix. 3,4‐dihydroxyphenylacetic acid, the only hydroxyphenylacetic acid identified in this study, has been previously reported in date seed aqueous extracts (Shi et al. 2023) and palm fruits (Ma et al., 2019).

##### 3.4.1.4 Hydroxyphenylpentanoic acids

Compound 16 was characterized as dihydroferulic acid 4‐sulfate. It was observed in both positive and negative ionization modes ([M − H]^−^ *m/z* 275.0205). Further characterization revealed product ions at *m/z* 195, 151, and 177, representing the removal of glucuronide (176 Da), C_8_H_8_O (120 Da), and C_6_H_6_O (94 Da), respectively (Sasot et al. 2017; Zeng et al. 2018). This compound was identified in KH‐DA, ZA‐ME, BA‐DA, and TH‐ME samples and previously reported in date palm pulp (Shi et al. 2023).

Compound 17, detected in positive mode at *m/z* 223.0973, was preliminarily characterized as 5‐(3',4',5'‐trihydroxyphenyl)‐γ‐valerolactone. Previously, Shi et al. (2024) reported the presence of this compound in all ripening stages of various date seed cultivars (including Zahidi, Deglet nour, Medjool, Thoory, Halawi, Barhee, Khadrawy, and Bau Strami), using ultrasound‐assisted extraction at low temperatures. However, in the current investigation, this compound was only detected in lightly roasted Zahidi date seed beverages, highlighting the sensitivity of this compound to heat.

#### 3.4.2 Flavonoids

The main class of phenolic compounds in date seed beverages was flavonoids (*n* total = 40), which included eight anthocyanins, seven flavanols, four flavanones, nine flavones, six flavonols, and six isoflavonoids.

##### 3.4.2.1 Anthocyanins

Anthocyanins were relatively less abundant in date seed beverage samples. Petunidin 3‐*O*‐(6''‐acetyl‐glucoside), identified as Compound 19, exhibited at *m/z* 522.1389 and pelargonidin 3‐*O*‐rutinoside, identified as Compound 21, exhibited at *m/z* 580.1740 were detected in KH‐LI. Petunidin 3‐*O*‐(6''‐acetyl‐glucoside) has been reported in wine and sweet cherry as noted by McDougall et al. (2005) and Hu et al. (2021).

Peonidin 3‐*O*‐diglucoside‐5‐*O*‐glucoside, identified as Compound 20, exhibited at *m/z* 788.2381, detected in ZA‐LI and BS‐ME samples, has been previously reported in brown seaweed (Lee et al. 2024).

Cyanidin 3,5‐*O*‐diglucoside (Compound 22, *m/z* 612.1662) and Cyanidin 3‐*O*‐(6''‐*p*‐coumaroyl‐glucoside) (Compound 23, *m/z* 596.1511) were detected in ME‐LI. Both compounds have been previously reported in kiwifruit (Zhu et al. 2021).

Cyanidin 3‐*O*‐(6''‐malonyl‐3''‐glucosyl‐glucoside) (Compound 24, *m/z* 698.1686), detected in medium‐ and dark‐roasted Halawi samples, was previously reported by Shi et al. (2024) in fresh Halawi date seeds at the *Tamar* stage.

##### 3.4.2.2 Flavanols

A total of seven flavanols were tentatively identified in date seed beverages. Procyanidin dimer B7 and Procyanidin trimer C1 were widely distributed across cultivars and roasting levels. Compound 28 (RT = 26.259 min with *m/z* 303.0876) was preliminarily characterized as 3'‐*O*‐methylcatechin. The presence of this compound was further characterized by product ions at *m/z* 271 and 163, representing the loss of a CH_3_OH and a C_6_H_5_O_2_, respectively (Reed 2009). This compound was found in medium‐ and dark‐roasted Medjool and dark‐roasted Halawi samples and reported by Subbiah et al. (2021) in blueberries.

Procyanidin dimer B7 (Compound 30, *m/z* 577.1318), detected in 17 samples across all roasting levels, exhibited a product ion at *m/z* 451. This compound has been identified in multiple lentil cultivars (Xia et al. 2023).

Compound 31 was preliminarily characterized as Procyanidin trimer C1 *at* m/z 865.1928, detected in medium‐ and dark‐roasted samples, exhibited product ions at *m/z* 739, 713, and 695, representing the loss of 126 Da (heterocyclic ring fission), 152 Da (RDA), and H_2_O (Kammerer et al. 2004).

##### 3.4.2.3 Flavanones

Narirutin (Compound 33, [M − H]^−^ *m/z* 579.1726) was confirmed by its product ions at *m/z* 271. This compound, previously identified in passion fruit, has been associated with potential chronic disease prevention properties (Liu et al. 2024).

Compound 34 was preliminarily characterized as naringin 4'‐*O*‐glucoside, displaying an [M − H]^−^ *m/z* of 741.2244. Its identification was supported by product ions at *m*/*z* 433 and 271, corresponding to the elimination of a rhamnose plus glucose moiety (308 Da) and the loss of a rhamnose plus two glucose moieties (470 Da), respectively (Yang et al. 2016). This compound, previously reported in citrus, has demonstrated strong antioxidant properties (Alam et al. 2014).

Compound 35 (RT = 57.904 min with *m/z* 341.1402) was characterized as 8‐prenylnaringenin. This compound was exclusively detected in the BS‐ME sample, and its identification was confirmed by MS/MS product ions at *m*/*z* 323, 271, and 137, corresponding to the loss of H₂O, a C₅H₉ fragment, and retro‐Diels–Alder (RDA) cleavage, respectively (Yu et al. 2020).

##### 3.4.2.4 Flavones

A total of nine flavones were identified, representing the most abundant flavonoid subclass. Apigenin 7‐*O*‐(6''‐malonyl‐apiosyl‐glucoside) (Compound 38) was detected in the [M − H]^−^ mode at *m/z* 649.1379 from dark‐roasted Khadrawy and Halawi samples. Its identification was further supported by the presence of a characteristic product ion at *m/z* 605. This compound has previously been reported in cumin (Ali et al. 2021).

Compound 44 was preliminarily identified as neodiosmin, detected in positive ionization mode with an [M + H]⁺ *m*/*z* of 609.1718. Its identification was supported by product ions at *m*/*z* 301 and 286. Neodiosmin has previously been reported in certain date palm cultivars during the early stages of ripening (Shi et al. 2024).

##### 3.4.2.5 Flavonol

Compound 46 (RT = 4.076 min with *m/z* 477.0678) was detected in both ME‐DA and HA‐DA samples in negative mode. This compound was preliminarily characterized as quercetin 3'‐*O*‐glucuronide, confirmed by the product ion at *m/z* 301. This compound was previously identified in lemon and mint and has been reported to protect cell membranes against lipid peroxidation due to its catechol structure (Chou et al. 2021).

Quercetin 3'‐sulfate (Compound 47) was identified with [M − H]^−^ *m/z* at 380.9938 Its identification was further supported by a product ion at *m/z* 79, corresponding to the loss of a sulfate group (SO₃, 80 Da; Kleinenkuhnen et al. 2019).

Compound 48 was tentatively characterized as Quercetin 3‐*O*‐rutinoside at *m/z* 609.1444), detected in four cultivars (mostly in dark‐roasted samples), was the most prevalent flavonol. This compound exhibited product ions at *m/z* 447, 463, and 301.

Compound 51 was preliminarily characterized as quercetin 3‐*O*‐(6″‐malonyl‐glucoside) 7‐*O*‐glucoside and was only found in sample ME‐LI. This compound was detected only in positive mode with [M + H]^+^ *m/z* at 713.1550 and further confirmed by the product ion at *m/z* 445, 300, and 169. Although most flavonols were predominantly found in medium‐ or dark‐roasted samples, this compound was uniquely detected in lightly roasted samples.

##### 3.4.2.6 Isoflavonoids

Compound 52 (RT = 12.510 min with *m/z* 533.1208), 53 (RT = 12.966 min with *m/z* 489.1358), and 54 (RT = 16.446 min with *m/z* 271.0606) were tentatively identified as 6''‐*O*‐malonylglycitin, 6''‐*O*‐acetylglycitin, and 3'‐hydroxydaidzein, respectively. These compounds were all detected with [M + H]^+^ *m/z* at 533.1308, 489.1358, and 271.0606. The existence of these compounds was previously reported by Shi et al. (2024) in various cultivars of date seed. Compound 55 was preliminarily identified as 5,6,7,3',4'‐pentahydroxyisoflavone and detected only in positive mode with [M + H]^+^ *m/z* at 303.0502 and further confirmed by the product ion at *m/z* 285 and 257. According to Peng et al. (2019), this compound was also found in mango peel.

#### 3.4.3 Other Polyphenols

A total of 12 phenolic acids were identified in this research, which included one alkylmethoxyphenol, one furanocoumarin, one hydroxybenzoketone, two hydroxycoumarins, one hydroxybenzaldehyde, one hydroxyphenylpropene, and five lignans.

Compound 59 was tentatively identified as furanocoumarins according to the precursor ion [M + H]^+^ *m/z* at 247.0606 and was only found in sample DE‐LI. The identity of furanocoumarins was further confirmed by the product ions at *m/z* 232, 217, 205, and 203. Compound 60 (RT = 4.122 min with *m/z* 261.0059) was preliminarily characterized as 2‐hydroxy‐4‐methoxyacetophenone 5‐sulfate confirmed by the fragment ions at *m/z* 181 and 97, representing the loss of [M‐H‐OSO_3_]^−^ and HSO_4_^−^, respectively (Pan and Cheng 2006). This compound was only found in date seed beverages made from dark roasting seeds and was previously found in *Ecklonia radiata* reported by Duan et al. (2024).

Lignans comprised the most abundant group of other polyphenols, with most compounds detected in medium‐ or dark‐roasted samples. Schisandrin (Sample 65) was found in the [M + H]^+^ mode at *m/z* 433.2231, further verified by product ion at *m/z* 415, 384, and 361. Schisandrin B (Compound 68) was detected in both ionization modes with [M + H]^+^ mode at *m/z* 401.1948, further confirmed by product ion at *m/z* 386. These two compounds were only found in medium‐roasted date seed beverages. Compound 69 was detected in both positive and negative mode with [M + H]^+^ at *m*/*z* 385.1651 which was tentatively characterized as schisandrin C based on the product ions produced at *m*/*z* 370, 315, and 300. This compound was found in TH‐DA, TH‐ME, and KH‐DA samples. This compound has been previously reported in ginger and lemon tea (Chou et al. 2021).

### 3.5 Phenolic Compound Distribution

**FIGURE 3:**
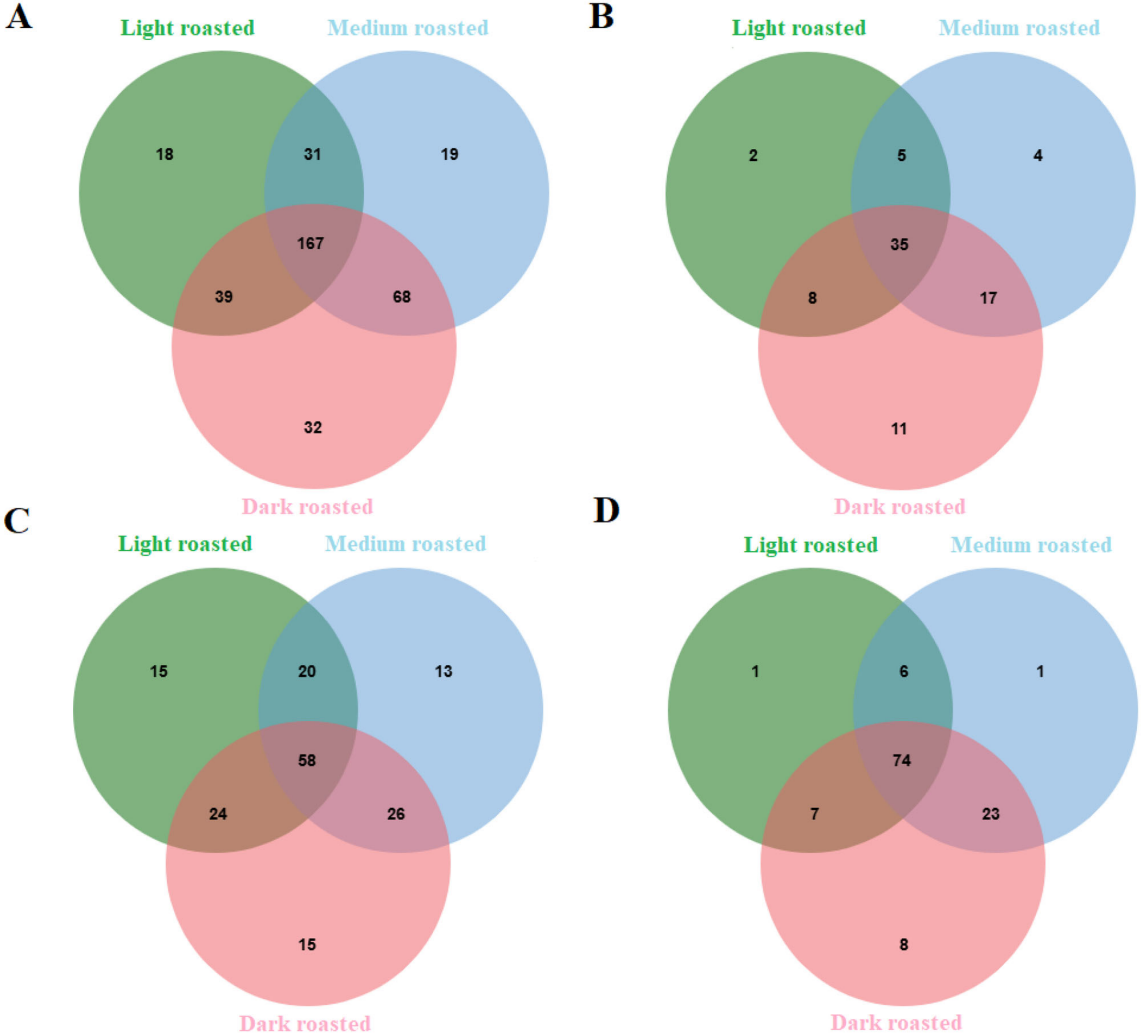
Venn diagram of phenolic compounds in date seed beverages at different roasting levels: (A) shows the comparison of total phenolic compounds at different roasting levels; (B) shows the comparison of total phenolic acids in different roasting levels; (C) shows the comparison of flavonoids in different roasting levels; and (D) shows the comparison of other phenolic compounds in different roasting levels.

Venn diagrams were created to visualize the overlap and uniqueness between different phenolic compound types and roasting levels (Figure 3). In total, 374 distinct phenolic compounds were identified in date seed beverage samples (Figure 3A). A total of 167 (about 44.65% of all the compounds) were shared across all three roasting levels (Figure 3A). In general, the number of detected compounds increased with increasing roasting temperature. Dark‐roasted samples had the highest number of unique compounds (32), followed by medium‐roasted (19) and light‐roasted (18) samples. The total types of phenolic compound also followed the similar trend.

The distribution of phenolic acids among the three roasting levels is shown in Figure 3B. A total of 35 (about 42.68% of all phenolic acids) compounds were detected in all three roasting levels of date seed beverage samples. Similar to the distribution of all phenolic compounds, the diversity of phenolic acids increased with increasing roasting intensity. Dark‐roasted samples had the highest number of unique phenolic acids (11), followed by medium‐roasted (4) and light‐roasted (2) samples.

Figure 3C illustrates the distribution of flavonoids, the most abundant group of phenolic compounds detected in date seed beverage samples. A total 171 compounds were detected, with 58 flavonoids (33.91% of all flavonoids) shared by all three roasting levels. Light‐ and dark‐roasted samples had the highest diversity of flavonoids, with 15 unique compounds each.

The distribution of other phenolic compounds is illustrated in Figure 3D. The dark‐roasted samples had the highest diversity, with eight unique compounds (about 6.67%). The largest overlap was observed between medium‐ and dark‐roasted samples (23 types; about 19.2%), followed by light‐ and dark‐roasted (seven types) and light‐ and medium‐roasted (six types) samples. Overall, these findings suggest that increasing roasting intensity enhances the diversity of the phenolic compound profile in date seed beverages.

The findings of this research highlighted that flavonoid constituted the most abundant group of phenolic compounds in date seed beverages, followed by phenolic acids and then other phenolic compounds. A typical trend observed across all three groups was an increase in the diversity of phenolic compounds with increasing roasting intensity. This increase may be attributed to two factors. Higher roasting temperatures may facilitate the release of a wider range of phenolic compounds from the date seed matrix into the brewing water. High temperatures may potentially induce structural changes in phenolic compounds, such as isomerization or decomposition, leading to the formation of new compounds. This phenomenon has been previously reported by Maghsoudlou et al. (2019). Although increased roasting resulted in greater phenolic compound diversity, a decline in overall antioxidant capacity was identified, as observed in the antioxidant assays. Therefore, careful temperature control is crucial to optimize roasting conditions, maximizing the release of beneficial compounds while minimizing the potential for thermal degradation and loss of antioxidant activity.

## 4 Conclusion

This study comprehensively investigated the antioxidant properties and phenolic composition characterization of date seed coffee‐like beverage derived from eight Australian date palm cultivars roasted at three temperatures. The LC‐ESI‐QTOF‐MS/MS analysis of date seed beverages resulted in identification of 69 phenolic compounds. Our findings indicated a general decline in antioxidant capacity and phenolic content with increasing roasting temperature. Beverages from lightly roasted date seeds, particularly those from the Deglet nour cultivar, demonstrated the highest antioxidant capacity. Medium‐roasted beverages from Deglet nour, Zahidi, and Bau Strami also demonstrated strong antioxidant properties. In contrast, Bau Strami emerged as the most promising cultivar for dark‐roasted beverages, exhibiting the highest antioxidant activity. Future studies should examine preferred sensory characteristics (aroma, flavor, mouthfeel) in relation to nutritional profiles across different roasting levels and cultivars, as this consumer preference will be essential for commercial viability. In addition, detailed studies on how roasting temperature affects specific phenolic compounds and their antioxidant mechanisms should be conducted in parallel with sensory analysis to develop optimized products that balance health benefits with consumer appeal. The focus on both biochemical properties and sensory quality will be important for developing date seed beverages as a sustainable, health‐promoting coffee alternative.

## Author Contributions

**Linghong Shi**: methodology, software, conceptualization, investigation, validation, formal analysis, writing–original draft. **Kashif Ghafoor**: supervision, writing–review and editing. **Claudia Gonzalez Viejo**: supervision, writing–review and editing. **Sigfredo Augusto Fuentes Jara**: supervision, writing–review and editing. **Farhad Ahmadi**: data curation, visualization, supervision, writing–review and editing. **Hafiz A.R. Suleria**: conceptualization, investigation, supervision, funding acquisition, project administration, resources, writing–review and editing, validation.

## Conflicts of Interest

The authors declare no conflicts of interest.

## Supporting information




**Supporting Information Figure S1** Comparison of phenolic content and antioxidant assays for different roasting levels and cultivars of date seeds
