# Supplementary material for: Impact of Roasting Temperature on Antioxidant Activities and Characterization of Polyphenols in Date Seed Beverages From Different Cultivars
Source: J Food Sci. 2025 May 7;90(5):e70242. doi: 10.1111/1750-3841.70242 (PMC12057541; doi:10.1111/1750-3841.70242)
Supplement: Supplementary file 1 — Supporting Information Figure S1 Comparison of phenolic content and antioxidant assays for different roasting levels and cultivars of date seeds [file JFDS-90-0-s001.docx]

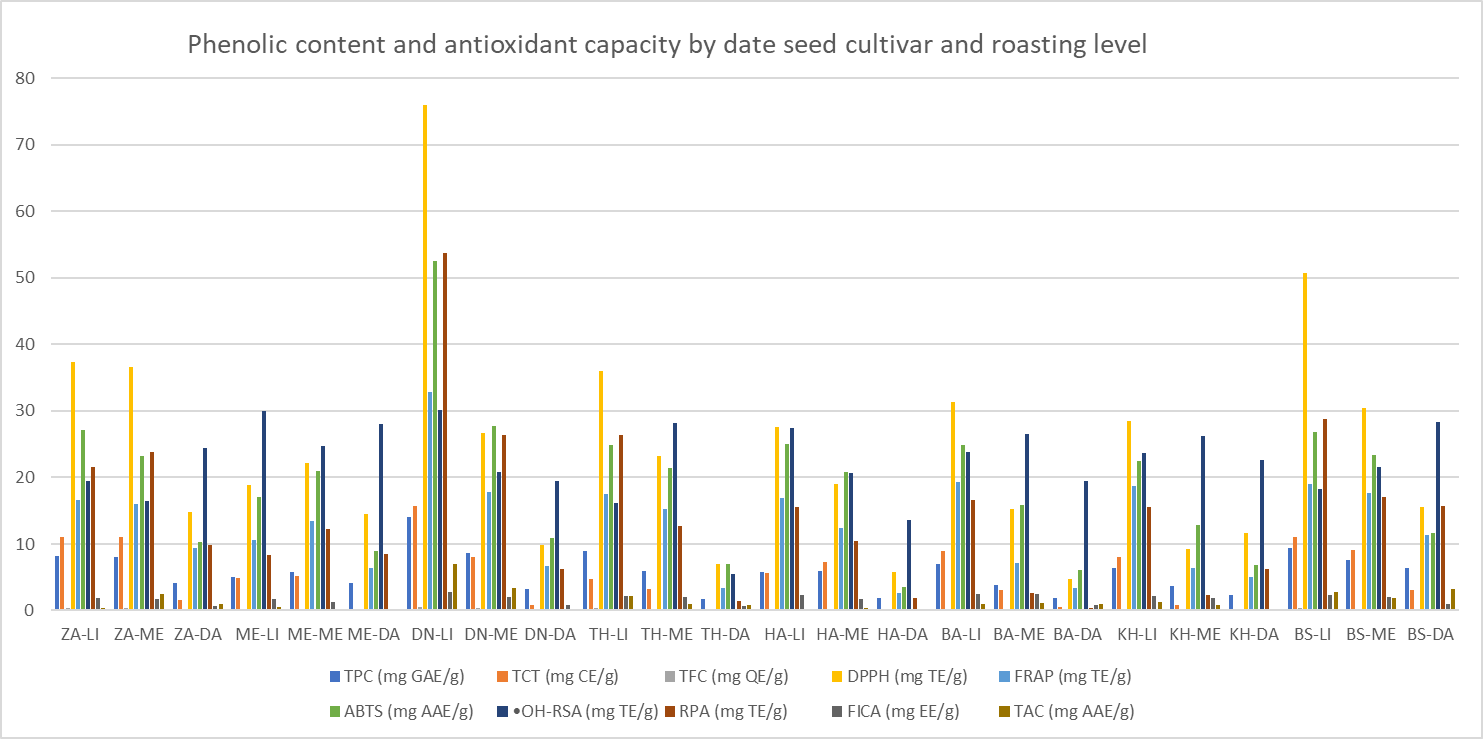


**Supplementary Figure S1.** Comparison of phenolic content and antioxidant assays for different roasting levels and cultivars of date seeds
